# Supplementary material for: Gender Differences in Association between Air Pollution and Daily Mortality in the Capital of the Green Lungs of Poland–Population-Based Study with 2,953,000 Person-Years of Follow-Up
Source: J Clin Med. 2020 Jul 23;9(8):2351. doi: 10.3390/jcm9082351 (PMC7464921; doi:10.3390/jcm9082351)
Supplement: Supplementary file 1 [file jcm-09-02351-s001.pdf]

Supplementary material

Table S1. Detailed mortality in Bialystok in the years 2008–2017.

	Total mortality (female)	Total mortality (male)	Cardiovascular mortality (female)	Cardiovascular mortality (male)	P
Total, N; mean age (SD)	16,154; 76.05 (15.48)	17,851; 68.33 (16.69)	8451; 81.29 (8.99)	7919; 72.18 (12.12)	<0.001
2008, N; mean age (SD)	1478; 74.10 (16.67)	1683; 67.33 (16.83)	750; 80.02 (9.34)	759; 71.86 (11.66)	<0.001
2009, N; mean age (SD)	1528; 74.61 (15.87)	1754; 67.23 (17.62)	797; 79.72 (9.44)	780; 72.12 (12.33)	<0.001
2010, N; mean age (SD)	1528; 74.47 (16.41)	1729; 67.67 (16.88)	841; 80.62 (8.45)	838; 71.16 (12.22)	<0.001
2011, N; mean age (SD)	1561; 74.47 (16.63)	1733; 68.14 (16.53)	817; 80.87 (9.39)	774; 72.95 (12.08)	<0.001
2012, N; mean age (SD)	1539; 75.80 (15.38)	1716; 67.84 (16.72)	836; 79.98 (9.9)	771; 71.91 (13.18)	<0.001
2013, N; mean age (SD)	1565; 77.07 (14.62)	1789; 67.79 (16.69)	844; 82 (8.46)	787; 71.06 (11.8)	<0.001
2014, N; mean age (SD)	1633; 77.43 (14.57)	1805; 68.84 (16.26)	909; 82 (8.22)	807; 72.88 (11.71)	<0.001
2015, N; mean age (SD)	1707; 76.72 (15.09)	1914; 69.2 (16.54)	849; 81.78 (8.99)	826; 72.52 (11.98)	<0.001
2016, N; mean age (SD)	1769; 76.97 (14.81)	1786; 69.4 (16.46)	877; 82.32 (9.46)	738; 72.66 (11.61)	<0.001
2017, N; mean age (SD)	1846; 78.08 (14.40)	1942; 69.59 (16.29)	931; 83.43 (8.39)	839; 72.72 (12.63)	<0.001

Table S2. Statistics for yearly concentrations of air pollutants and weather conditions in the period of 2008–2017.

	NO_2 µg/m³	SO_2 µg/m³	$PM_{2.5}$ µg/m³	PM_{10} µg/m³	Temp. °C	RH %	Atm. P. hPa
No. of observations, N, %	3620, 99.09%	3597, 98.45%	2938, 80.43%	3248, 88.91%	3653,1 00%	3653, 100%	3653, 100%
Annual mean–2008	14.51	2.64	N/D	23.92	8.21	81.90	997.02
Annual mean–2009	14.37	1.56	18.83	25.95	7.22	83.22	996.56
Annual mean–2010	15.16	3.61	23.94	27.82	6.80	81.221	995.22
Annual mean–2011	16.04	3.76	20.85	27.83	7.61	83.71	998.74
Annual mean–2012	14.54	3.32	22.29	27.79	7.13	82.08	996.94
Annual mean–2013	14.41	3.22	19.29	22.90	7.64	79.04	997.09
Annual mean–2014	13.66	4.24	21.90	24.89	8.17	77.62	998.11
Annual mean–2015	14.87	4.11	21.31	25.01	8.73	79.69	998.90
Annual mean–2016	13.46	3.17	17.48	19.78	8.04	81.33	997.41
Annual mean–2017	13.09	3.46	17.69	21.06	7.94	78.35	996.72
Exceeded annual mean WHO guidelines values (number of years)	0	N/A	10	10	N/A	N/A	N/A

Abbreviations: Atm. P., atmospheric pressure; IQR, interquartile range; ***N/A***, not applicable; N/D, no data; NO_2, nitrogen dioxide; ***$PM_{2.5}$***, particulate matter with a diameter of 2.5 µm or less; PM_{10} particulate matter with a diameter of 10 µm or less; ***RH,*** relative humidity; SD, standard deviation; SO_2, sulfur dioxide; Temp., temperature; WHO, World Health Organization.

Table S3. Spearman's correlations between physical variables and air pollutants.

NO_2 µg/m³—daily mean (N=3620)	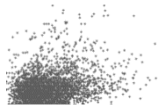	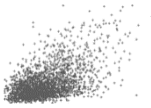	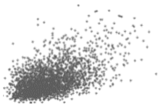	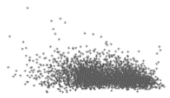	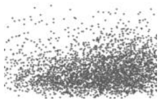	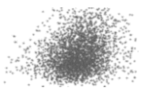
R=0.28 P<0.001	SO_2 µg/m³—daily mean (N=3597)	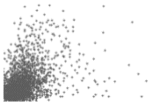	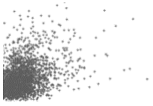	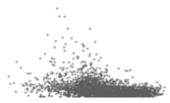	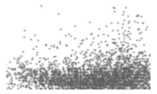	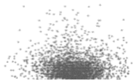
R=0.53 P<0.001	R=0.45 P<0.001	$PM_{2.5}$ µg/m³—daily mean (N=2938)	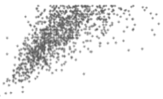	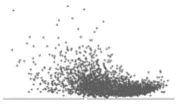	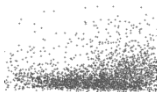	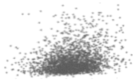
P=0.49 P<0.001	R=0.40 P<0.001	R=0.84 P<0.001	PM_{10} µg/m3—daily mean (N=3240)	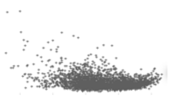	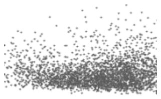	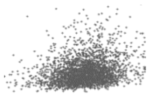
R=-0.41 P<0.001	R=-0.33 P<0.001	R=-0.47 P<0.001	R=-0.32 P<0.001	Temp. °C daily mean (N=3653)	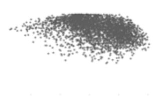	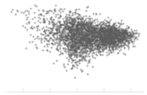
R=0.14 P<0.001	R=0.09 P<0.001	R=0.17 P<0.001	R=0.03 P=0.08	R=-0.36 P<0.001	RH, % daily mean (N=3653)	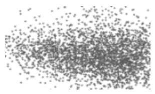
R=0.22 P<0.001	R=0.06 P=0.002	R=0.22 P<0.001	R=0.22 P<0.001	R=-0.26 P<0.001*	R=-0.19 P<0.001*	Atm. P. hPa daily mean (N=3653)

Abbreviations: Atm. P., atmospheric pressure; NO_2, nitrogen dioxide; ***$PM_{2.5}$***, particulate matter with a diameter of 2.5 µm or less; PM_{10}, particulate matter with a diameter of 10 µm or less; RH, relative humidity; SO_2, sulfur dioxide; Temp., temperature.
